# A novel tablet-based motor coordination test performs on par with the Beery VMI subtest and offers superior temporal metrics: findings from children with pediatric acute-onset neuropsychiatric syndrome

**DOI:** 10.1007/s00221-023-06612-x

**Published:** 2023-04-13

**Authors:** Max Thorsson, Martyna A. Galazka, Parisa Hajjari, Elisabeth Fernell, Jonathan Delafield-Butt, Christopher Gillberg, Mats Johnson, Jakob Åsberg Johnels, Nouchine Hadjikhani

**Affiliations:** 1 Gillberg Neuropsychiatry Centre, Institute of Neuroscience and Physiology, Sahlgrenska Academy, Gothenburg, Sweden; 2 Laboratory for Innovation in Autism, University of Strathclyde, Glasgow, Scotland, UK; 3 Section of Speech and Language Pathology, Institute of Neuroscience and Physiology, Sahlgrenska Academy, Gothenburg, Sweden; 4 Athinoula A. Martinos Center for Biomedical Imaging, Massachusetts General Hospital, Boston, MA USA; 5 Gothenburg, Sweden

**Keywords:** Motor assessment, Visual motor skill, Neurodevelopmental disorders, PANS, Tablet-based testing

## Abstract

Neuropsychiatric and neurodevelopmental disorders are often associated with coordination problems. Pediatric Acute-onset Neuropsychiatric Syndrome (PANS) constitutes a specific example of acute and complex symptomatology that includes difficulties with motor control. The present proof-of-concept study aimed at testing a new, bespoke tablet-based motor coordination test named *SpaceSwipe*, providing fine-grained measures that could be used to follow-up on symptoms evolution in PANS. This test enables computationally precise and objective metrics of motor coordination, taking into account both directional and spatial features continuously. We used *SpaceSwipe* to assess motor coordination in a group of children with PANS (*n* = 12, assessed on in total of 40 occasions) and compared it against the motor coordination subtest from the Beery-Buktenica Developmental Test of Visual-Motor Integration (Beery VMI) 6th edition, traditionally used to follow-up symptomatology. Using a bivariate linear regression, we found that 33 s of the directional offset from tracking a moving target in *SpaceSwipe* could predict the Beery VMI motor coordination (VMI MC) raw scores (mean absolute error: 1.75 points). Positive correlations between the predicted scores and the VMI MC scores were found for initial testing (*r*_adj_ = 0.87) and for repeated testing (*r*_adj_ = 0.79). With its short administration time and its close prediction to Beery VMI scores, this proof-of-concept study demonstrates the potential for *SpaceSwipe* as a patient-friendly tool for precise, objective assessment of motor coordination in children with neurodevelopmental or neuropsychiatric disorders.

**Supplementary Information:**

The online version contains supplementary material available at 10.1007/s00221-023-06612-x.

## Introduction

### Motor coordination problems in children with PANS

Symptoms related to motor control and coordination are well documented in children with Pediatric Acute-onset Neuropsychiatric Syndrome (PANS) (Murphy et al. 2015a, b; Orefici et al. 2016), which is defined as the highly disabling sudden onset of neuropsychiatric symptoms, specifically obsessive–compulsive symptoms/obsessive–compulsive disorder and/or eating restrictions, combined with other behavioral and neurological changes, including motor difficulties.

Difficulties with motor control in children with PANS include clumsiness, motor hyperactivity, tics (movement without purpose), or choreiform (involuntary, random, and non-rhythmic) movement (Swedo 2012), as well as sudden difficulties with handwriting. These difficulties can hinder a child’s development, making it difficult to participate in meaningful social activities (Gillberg, Harrington, and Steinhausen, 2006), while also increasing the risk of being bullied (Bejerot et al. 2011).

One specific aspect of motor issues in PANS relates to difficulties with fine motor coordination, which can affect skills such as feeding, dressing, handwriting, or drawing. For example, Colvin et al. (2021) identified graphomotor difficulties in children with PANS*,* using the Beery-Buktenica Developmental Test of Visual-Motor Integration (Beery VMI) motor coordination subtest (VMI MC), while Lewin et al. (2011) found that more than two-thirds of parents reported that their children with PANS had difficulties with handwriting. Other than writing difficulties, problems in copying complex figures have been observed when children with PANS were administered the Rey-Osterrieth Complex Figure (ROCF). Writing and drawing have indeed been proposed as useful for the evaluation of motor symptoms in PANS (Swedo 2012). Still, it is important to keep in mind that judgments of the quality of drawings and handwriting are subjective, and that important aspects related to motor timing can be missed with a pen-and-paper test—such as most of the temporal features, which are clearly important aspects of the prospective organization motor control (Bucsuházy and Semela 2017; Delafield-Butt et al. 2018; Elliott et al. 2010; Lamb et al. 2016; Lin et al. 2015; Hofsten 2004). Computationally precise, continuous, and objective measures of fine spatiotemporal motor control are needed to better understand disruption in motor control, such as their sub-second temporal organization, which is not always observable by human-rated instruments or in the final product of a movement [e.g., as in the computational characterization of motor signatures that appear to underpin motor disruption in autism spectrum disorder in Anzulewicz et al. (2016), Chua et al. (2022), Torres et al. (2013) and Torres (2013)].

Moreover, co-occurring disorders can make findings from motor assessment difficult to interpret (Blank et al. 2019), and several factors can influence or hinder motor coordination testing when assessing children with neuropsychiatric symptoms. For example, difficulties with attention may limit a feasible testing situation to very short periods of time, while specific difficulties related to obsessive–compulsive symptoms or anxiety may completely hinder the assessment. Importantly, since most children have a combination of difficulties (Gillberg 2010), reliable motor testing can be impossible using traditional assessments. Given this, there seems to be a real need to find an objective and efficient test of fine motor performance.

### Traditional motor coordination tests

Assessment of motor control is complex. Several instruments have been developed and standardized on a very large number of individuals to evaluate motor coordination in children (Cancer, Minoliti, Crepaldi and Antonietti 2020). One clinical standard instrument developed specifically for manual motor coordination and visual-motor integration is the Beery VMI (Crotty and Baron 2011). The VMI MC consists of 3 questions about motor development, 3 imitation tracing paths, and 24 paths that the child traces by themselves. To complete the task, the child is required to trace a figure with a pencil without drawing outside the borders of the paths. The cut-off percentile for the Beery VMI used in research for Developmental Coordination Disorder (DCD) (American Psychiatric Association 2017) varies depending on the specific study and the population being studied. Performance below the 25th percentile (Beery 2010; Lahav et al. 2013; Valverde, Ribeiro Soares Araújo, Magalhães and Cardoso 2020) has been suggested as the cut-off point for below-average motor performance, which is higher than the 15th and 16th percentile cut-off used in research for indicating severe difficulties with motor coordination that impact daily life [e.g., Ghayour Najafabadi et al. (2022); Smits-Engelsman et al. (2015)].

The findings examining the relationship between writing performance and the VMI MC are conflicting. For example, when it comes to temporal aspects, several research efforts have failed to correlate writing speed, such as the number of letters written per minute, with VMI MC scores (Brown and Link 2015; Volman et al. 2006; Duiser et al. 2014), while others (Rosenblum, Amit Ben Simhon, Meyer and Gal 2019) found a significant, but weak relationship between the pen stroke time on paper and the VMI MC when testing children diagnosed with autism.

More comprehensive assessments are time-consuming, take up to one hour to complete including manual scoring (e.g., Bruininks-Oseretsky Test of Motor Proficiency Second Edition (Lazaro, Reina-Guerra, Quiben, and Umphred 2020) and Movement Assessment Battery Second Edition [MABC-2] (Brown and Lalor 2009; Lazaro et al. 2020)), and require considerable clinical expertise. Additionally, these assessments (including the VMI MC) primarily depend on discrete scoring systems, such as the number of errors or total time limits, rather than a continuous evaluation of the movement execution. Shorter test batteries can be informative about neurodevelopmental delays but suffer from some of the same drawbacks (Gillberg et al. 1983). The functional aspects such as directional and spatial features are seldom measured in combination with temporal accuracy, which is problematic from an ecological validity perspective, as both spatial and angular accuracy are required when performing multitudes of daily tasks. Distinguishing directional and spatial aspects of movement is critical in identifying mechanisms that underlie them (Graaf et al. 1994; Lussanet et al. 2002). Spatial control is the product of movement execution, which involves joint angular movement (Morasso 1981), supported by the cerebellum and basal ganglia interconnection (Bostan et al. 2010; Manto 2009; Rolls and Treves 1997; Todorov et al. 2019). Difficulties with spatial control are an important feature to consider when assessing motor control. Directional features provide more specific information about the angular quality of the movement production.

In considering both the functional and practical aspects, it is evident that, in order to identify and understand motor control difficulties in children with neuropsychiatric problems, including those with PANS, motor testing needs to be accessible, attractive for the children, and also, importantly, time-efficient, so as not to be burdensome for the clinician or researcher, or the child. Moreover, we argue that such tests need to assess the kinematic and temporal features of moment-by-moment control with continuous metrics, and include sub-second data collection that allows for the calculation of specific motor control features, such as directional and spatial accuracy in relation to motor timing. Finally, it is also critical to consider the child’s age. As children get older, their motor skills predictably improve. For children with PANS, however, motor skills development is characterized by an abrupt regression (Swedo 2012), making it less clear how aspects of motor control and age are related in this population.

### Novel tablet-based tests

New technological advances in touch-screen technology allow for high frame-rate data collection, using a medium that children are familiar with, and often like. In fact, research has shown that tablet-based tests have the potential to eventually replace paper-based tests (Bignardi et al. 2021). For example, the tablet-based ROCF has been successfully implemented in adolescents (Hyun et al. 2018) and adults (Savickaite, Morrison, Lux, Delafield-Butt and Simmons 2022), with the noticeable addition of features related to the timing of the task execution. Another example is Howe et al. (2017) who presented a computerized perceptual-motor skills assessment, which included paths similar to the VMI MC, but on a tablet using a stylus. They found that the error of traced paths administered on a tablet correlated with the standard Developmental Test of Visual Perception Second Edition (Hammill, Voress and Pearson 2004). Gerth et al. (2016) investigated some of the paths of the VMI on a tablet with a stylus and compared them to their performance with pen-and-paper. The children’s performance in scores was significantly different, and in addition, yielded a clear ceiling effect.

Computerized solutions can also be used to investigate continuous aspects of movement, such as done by Culmer et al. (2009), who presented a motor test on a computer screen using a stylus and that included copying, aiming, and tracking tasks.

Others have used finger touch-based solutions [e.g., Chua et al. (2022); Lu et al. (2022); Matic and Gomez-Marin (2019)]. For example, Anzulewicz et al. (2016) developed a novel application and showed the usability of portable tablets and their internal sensors to measure finger-based swipes in younger autistic children. Chua et al. (2022) and Lu et al. (2022) measured spatiotemporal kinematics of displacement toward a static target. Matic and Gomez-Marin (2019) also used this medium employing a custom Android application, and investigated hand movement constraints, such as slowing down with increasing curvature as well as a task for tracking a moving target. Other studies that have been implementing tracking (Culmer et al. 2009; Hill et al. 2016), were testing trajectories of constant speed and did not consider the smoothness of movement profiles that occurs in well-controlled curvilinear movement (Huh and Sejnowski 2015; Viviani and Schneider 1991; Zago et al. 2018).

Further, none of the tests developed so far have been attended to analyze both directional (movement direction) and spatial (movement position) aspects of continuous fine motor displacement. As argued above, we consider that both metrics are required for assessing motor coordination.

In addition, here, we sought to improve the hand movement ergonomics of tablet-based motor coordination testing, with a bespoke device that includes a purpose-built game to address important adjustments needed for children with neuropsychiatric and neurodevelopmental problems (such as sound sensitivity, communication impairments, and attention difficulties).

Finally, it is important to consider that the quality of motor control is not simply the result of the speed of the movement execution. Motor control also concerns the entire movement execution, such as continuously redirecting and repositioning one’s movement accordingly throughout the task. Therefore, motor control should be quantified with metrics that inform about the timing of directional and spatial features of motor coordination. As previously discussed, tablet-based tests have the advantage to permit detailed analyses of parameters that are not easily captured using traditional instruments.

### The current study and feasibility of a novel tablet-based test

In the current proof-of-concept study, we introduce a touch screen-based system that we named *SpaceSwipe*, consisting of an ergonomic, task-dedicated device, and including adjustments specifically developed for individuals with neurodevelopmental/neuropsychiatric disorders.

*SpaceSwipe* is based on the task of tracking a moving target in nine different trajectories, with varying speed and path complexity, allowing the continuous evaluation of motor coordination over time. *SpaceSwipe* quantifies motor coordination 60 times per second, using directional and spatial offsets to a moving target, as opposed to traditional tests that are scored after execution, using total time limits. This way both the spatial and directional metrics include temporal accuracy. In addition, the varying speed of the trajectories allows assessment of how fast the movements can be performed accurately. The directional offset relates to how well the target movement direction was followed, and the spatial offset to how close the movement’s position was to the target movement position. While the VMI MC also measures spatial offset, it is only scored afterward, based on a maximum allowed distance from the target path. In contrast, the directional offset accounts for directional corrective visuomotor processes. To our knowledge, this metric has not been deployed before in this context, yet it can potentially provide more specific information about children’s neuromotor difficulties (Zwart et al. 2019) not readily accessible to current instruments. To evaluate the potential role of these novel metrics, three research questions (RQ) were addressed:

[RQ1] Can offset metrics to the moving target in *SpaceSwipe* predict the VMI MC score and are the results stable and reproducible?

[RQ2] Is there a relationship between the spatial and directional metrics in *SpaceSwipe* and the VMI MC score?

[RQ3] Is there a relationship between age and the spatial and directional metrics in *SpaceSwipe*?

Given previous findings, we expected a positive correlation between the skill of maintaining and adjusting direction in tracing a path with a pencil (as in the Beery VMI), and in tracking a moving target on a touch screen (as in *SpaceSwipe*). We further hypothesized that the directional offset from *SpaceSwipe* would be related to the VMI MC score, as both involve coordination of movement direction. The VMI MC score has previously not been related to temporal features of handwriting (Brown and Link 2015; Rosenblum et al. 2019; Volman et al. 2006), but we expected the metrics from slower trajectories to be most related to the VMI MC. Finally, since the VMI MC (Beery 2010) and tablet-based tracking test (Flatters, Hill, Williams, Barber and Mon-Williams 2014; Hill et al. 2016) results are known to relate to age, we expected that both the spatial and directional offsets to the moving target would similarly reduce with age.

## Methods

### Participants

The study was approved by the Swedish Ethical Review Authority guidelines. Participation was voluntary. All caregivers gave written consent for their child’s participation before the experiment, in addition to the child’s assent or consent, if appropriate. We examined a unique group of 12 children (6 girls, 6 boys) who all met research diagnostic criteria for PANS (median age 10 years [range: 5–16 years]), in order to evaluate the feasibility of this test on a population with a specific neuropsychiatric disorder. The participants took part in a larger, ongoing study examining the effect of Intravenous Immunoglobulin (IVIG) treatment, in which they received IVIG once monthly for 3 months, and thereafter single IVIG doses as needed, depending on symptom development, up to a maximum total of six IVIG treatments. The motor coordination test was performed four times in the course of the treatment (0, 3, 8, and 12 months). Five of the participants had been diagnosed with co-occurring neuropsychiatric diagnoses: attention-deficit/hyperactivity disorder (ADHD) [4], autism/Asperger syndrome [1], autistic-like condition [3], and unspecified epilepsy [1]. Another three participants had pre-existing neurodevelopmental symptoms but had not received a specific diagnosis. None of the children had been diagnosed with an intellectual disability or speech/language impairment. Demographics are shown in Supplementary Table 1. PANS symptoms were measured with the investigator-rated PANS scale, Pediatric Acute Neuropsychiatric Symptom Scale, Parent Version (Murphy and Bernstein 2012), covering the whole spectrum of symptoms included in the PANS diagnosis. At baseline, all participants had moderate to severe obsessive–compulsive symptoms. Other common symptoms were anxiety, emotional lability and depression, tics, irritability and aggressive behavior, behavioral regression, difficulties with attention and learning, sensory symptoms, motor symptoms, and specific sleep disturbances. Specific symptoms identified by the PANS scale are shown in Supplementary Table 2.

As per the IVIG treatment protocol, each child was asked to perform both motor coordination tests (*SpaceSwipe* and Beery VMI MC) on each of the four visits. The order in which these two tests were performed was counterbalanced across participants.

### SpaceSwipe properties

#### Task-dedicated device

A custom case for the device was designed in OpenSCAD (Kintel 2010) to robustly position the touch screen at an ergonomic angle for the wrists, with cut-outs to be able to fix the device to the table. A Raspberry Pi 3B + with a 7-inch LCD touch screen (480 × 800 pixels [pix]), was used to register the child’s active touch at 60 Hz. The touch screen was 155 mm wide, which made the pixel size, approximately 0.19 mm. Customized hardware was developed as a preference over commercial systems, such as iPad tablet computers, in order to assure a controlled test situation, by allowing for structured hand movement, and full insight into hardware specifics and software stability, through a Debian-based operating system.

#### Game directive and test setting

**Fig. 1:**
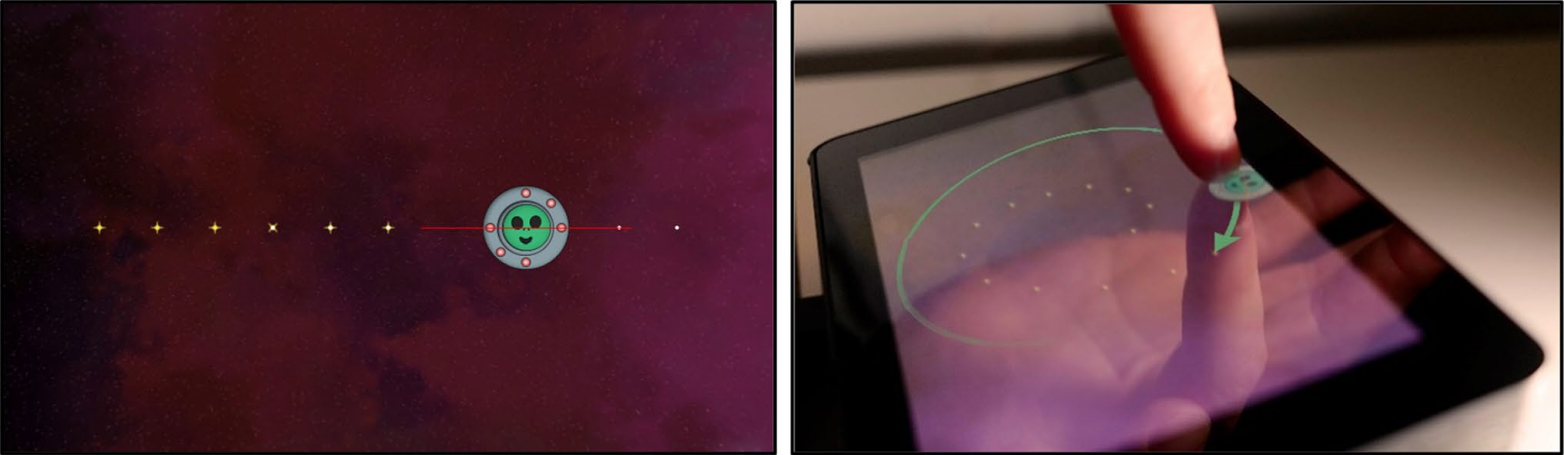
Left panel: Screen capture from a horizontal line trajectory in *SpaceSwipe*. Right panel: Example gameplay of *SpaceSwipe*. The green arrow is added to display the motion. Copyright © 2020 Max Thorsson

The task in *SpaceSwipe* is for the child to keep an alien inside a moving spaceship. The child gets positive feedback when he manages to hold the alien in the spaceship in the form of a green glow around the spaceship with stars lighting up along the target trajectory. A screen capture during *SpaceSwipe* and an example gameplay image are shown in Fig. 1.

**Fig. 2:**
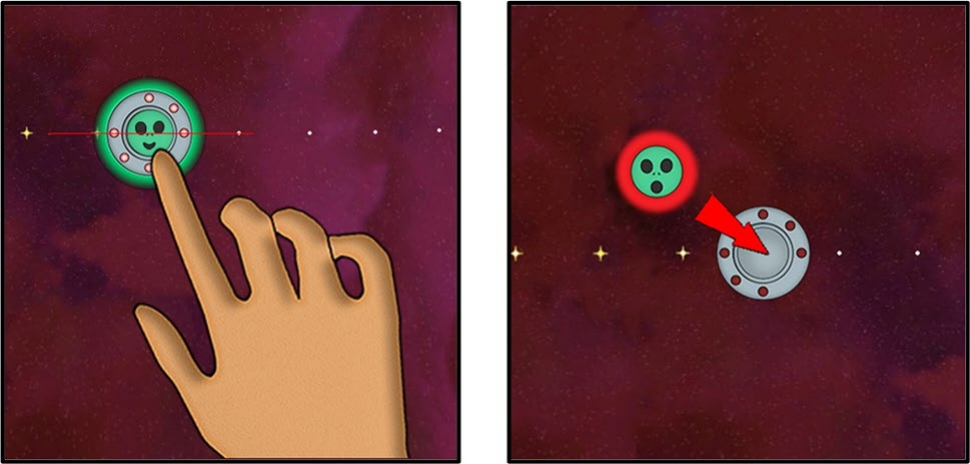
Images to display the game directive of *SpaceSwipe.* Left panel: Tracking of the spaceship. Right panel: If the alien was too far away an arrow showed the way back. Copyright © 2020 Max Thorsson.

The game is presented in small steps, with visual instructions to make it easy to understand. First, the experimenter reads the game instructions, with images that show the task (see Fig. 2). Second, an automated session with a cartoon hand displays the task of tracking the spaceship along a horizontal trajectory.

Third, the experimenter shows how to perform the same horizontal line. Finally, the participant is asked to try the same trajectory themselves. If the touch is too far away from the spaceship (> 9 mm away from the center of the ship) for the duration of 1.7 s, the game level resets. This was intended as a pedagogical way to motivate the child to follow and learn the task, as well as a confirmation that the child had understood the task.

In addition to the instructions, several adaptations were made to ensure that the game was best suited for children with difficulties. The game is presented in short intervals to allow the opportunity for the child to take a break if needed. Furthermore, the gameplay is by default silent, to minimize issues related to sound sensitivity. To facilitate ergonomics and natural hand movement, the child sits in a chair of the correct height, so that their elbows are approximately 90 degrees, and their wrists are in a comfortable position during the test.

*SpaceSwipe* was written in Python (Rossum 1995) using the PyGame, game development library (Lindstrom, René Dudfield, Shinners, Dudfield and Kluyver 2011). The sky graphics were generated from the online WebGL Space 3D generator (Terrel and Himbolt 2015). All other graphical elements (e.g., alien, spaceship, and graphical effects) were created by the first author (Copyright © 2020 Max Thorsson).

#### Path generation

The shape characteristics of the paths were generated from a periodic modification of a *normalized tunable sigmoid function*, which was used to represent an array of the tangential angle, that through the cumulative sum of the cosine and sine would create custom paths. The mathematical operations and our Python implementation to create the paths are explained in detail in the Supplementary Methods.

#### Motion profile generation

The motion profile for the straight lines was based on the minimum-jerk trajectory. The minimum-jerk trajectory is a smooth trajectory obtained by minimizing the jerk (change of acceleration) between two positions and has been proposed to be crucial for human upper limb movement (Flash and Hogan 1985). The tilted lines were at a constant speed and the motion profiles of the more complex paths were based on a power–law relationship between curvature and speed (Huh and Sejnowski 2015; Lacquaniti et al. 1983). Further details about our Python implementation for motion profile generation can be found in the Supplementary Methods.

#### Degree of difficulty

The path-shaping parameters and average speed of the trajectories were manually tuned by a physiotherapist, who has experience testing the motor abilities of children with neurodevelopmental/neuropsychiatric disorders. Nine different trajectories were chosen (horizontal line, right tilted line, left tilted line, as well as zigzag and spiral trajectories [at three different average speeds]). The idea was to start with simple trajectories and then increase both path complexity and speed. Both spiral and zigzag paths were included to investigate movement to trajectories with constant and alternating directions. The slower, more complex paths were intended to be like traditional tracing tasks as in the VMI MC, without the high demand for timing. All line trajectories were at the average speed of 100 pix/s. The zigzags and spirals were in 100 (A), 225 (B), and 300 (C) pix/s. Abbreviations, speeds, and durations of the trajectories are displayed in Table 1.

**Table 1:** Information about *SpaceSwipe’s* trajectories

Path type	Abbreviation	Speed (pix/s)	Speed (cm/s)	Duration (s)
Horizontal line	hline	100	0.20	4.27
Right tilted line	tlineR	100	0.20	4.27
Left tilted line	tlineL	100	0.20	4.27
Zigzag	zigzagA	100	0.20	10.50
Zigzag	zigzagB	225	0.43	4.65
Zigzag	zigzagC	300	0.57	2.98
Spiral	spiralA	100	0.20	22.46
Spiral	spiralB	225	0.43	10.47
Spiral	spiralC	300	0.57	6.75

#### Offset metrics

**Fig. 3:**
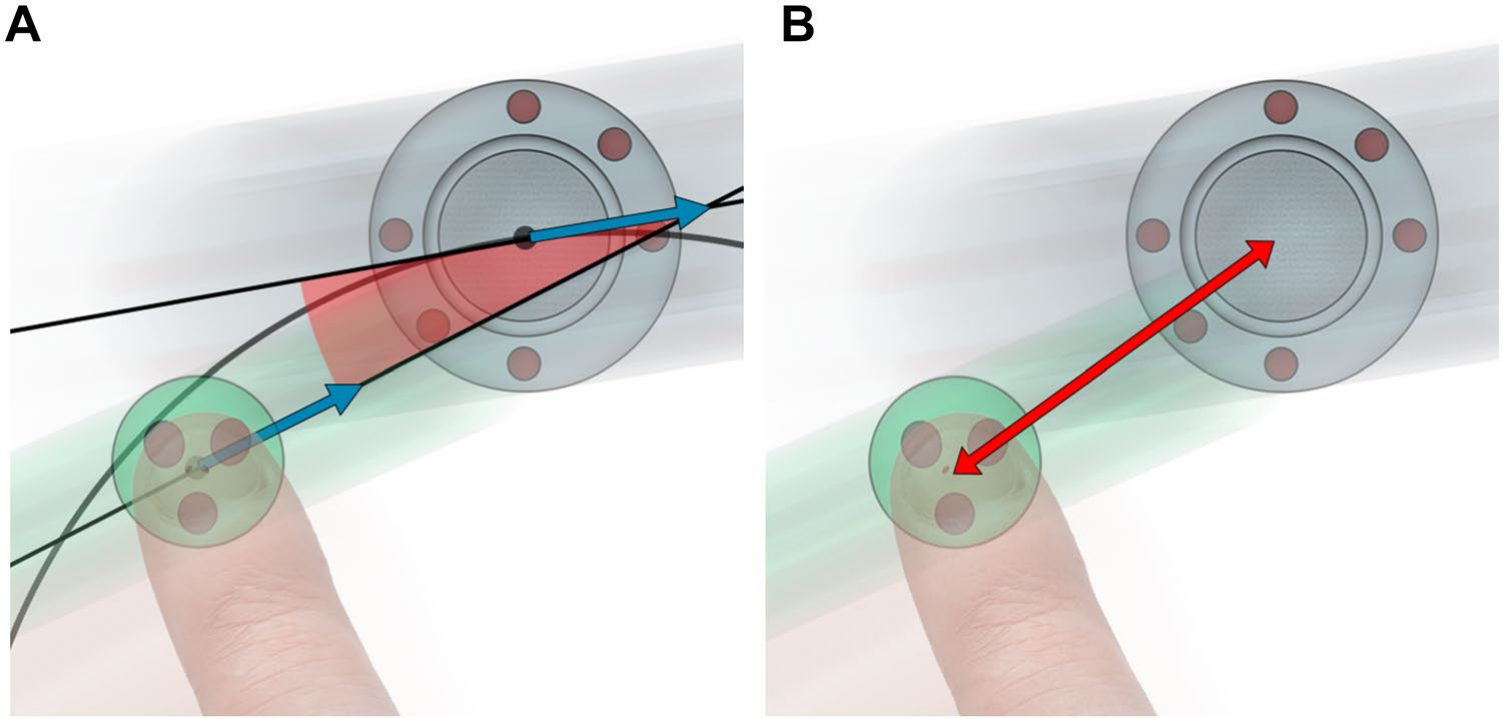
Measures of **A** directional and **B** spatial offset. **A.** The directional offset (red angle) is defined as the angle between the direction of the tangential angles from the spaceship (large gray circle) and the participant’s movements. **B.** The spatial offset (red line) is defined as the Euclidean distance between the participant’s fingertip (alien, small green circle) and the center of the moving spaceship

Motor performance was measured by offset metrics to the target movement’s position and direction. The *directional offset* was expressed as the delta between the tangential angles of the spaceship’s and the participant’s movements (see Fig. 3A). The *spatial offset* was expressed as the distance that the participant had to the target position, the Euclidean distance between the touch position and the spaceship center (see Fig. 3B). Mathematical operations for how the metrics were estimated are outlined in the Supplementary Methods.

#### Pre-processing of touch screen data

Touch screens, much like other electronic instruments, risk having internal noise. Therefore, we filtered the raw position (x- and y-touch screen coordinates) time series using a zero-shift Butterworth low-pass filter, which is common practice for filtering movement data (Bartlett 2007). The principle is to dampen the frequencies that are faster than the participant’s movements. We followed similar procedures recently implemented by Matic and Gomez-Marin (2019) and Chua et al. (2022) and used a cut-off of 8 Hz with a 4th-order filter. The smoothing obtained by the low-pass filter is also favorable for estimating tangential angles. In our case, we did not remove any suspected outliers due to the substantial risk of removing features such as “slipping” and “jerky” movement, which can be important markers for motor difficulties. The *SpaceSwipe* software was designed to be computationally inexpensive for stable data sampling. The mean standard deviation of the frame rate, based on all collected sessions was 1.7 ms, which for our purposes here, was negligible. Thus, we did not perform any resampling in our pre-processing of the data and assumed a constant frame rate for simplification of the calculations.

### Statistical analysis

Based on the theoretical assumption that the slower more complex paths would have similarities to traditional path tracing tasks as in the VMI MC, we chose the directional offsets from the two slowest most complex paths of *SpaceSwipe* (*zigzagA* and *spiralA*), to evaluate the prediction of the VMI MC score.

We first investigated if the chosen directional offset metrics to the moving target in *SpaceSwipe* could predict the VMI MC score for the initial testing. A bivariate linear regression, ordinary least squares (OLS), was fitted using the two directional metrics for predicting the VMI MC score. Because age and age squared (Beery 2010) are known to be related to motor development, we first wanted to make sure that they were controlled for, when analyzing the explanatory power (*R*^2^) of our predictors. Therefore, following the guidelines by Reid and Allum (2019) both age and age squared were included in the analysis. In order to control for the effect of age in predicting the VMI MC score, semi-partial (part) *R*^2^ was estimated, following the method described by Kim (2015).

We then examined whether the results were stable and reproducible by looking at repeated testing, to account for variability in the motor coordination in children with variable neuropsychiatric symptoms. Linear mixed-effects models (LMMs) were used, as they present the advantage of accounting for a repeated and variable number of data points per participant (Snijders and Bosker 2012), thereby estimating the composite nested effect of all data points. The participants were included as random effects for the intercepts [c.f., Wit and Buxbaum (2017); Geers et al. (2022); Runnalls et al. (2019); Stipancic et al. (2021)] and the two directional metrics were set as predictors for the VMI MC score. The significance of the fixed effects in the nested model was assessed with F-tests, using the Satterthwaite method for degrees of freedom approximation, as implemented in the r package lme4 (Bates et al. 2015). We used multiple data points per individual, to account for the variability of symptoms. Yet, caution is still needed when interpreting effect sizes, given the small number of children included.

Since LMMs have multiple levels, *R*^2^ could not be estimated identically as for OLS. Nakagawa and Schielzeth (2013) recommend that two types of *R*^2^ should be reported for LMMs (marginal and conditional *R*^2^), as both provide important complementary information. Marginal *R*^2^ is most similar to traditional *R*^2^ as it describes the proportion of variance explained by the fixed factor(s). Conditional *R*^2^ also includes the random effects, thus a model with very low marginal and high conditional *R*^2^ indicates that there is much-unexplained variance across individuals. We used the r-package, Part R2 (Stoffel, Nakagawa and Schielzeth 2021), to obtain confidence intervals marginal and conditional *R*^2^.

Further, to investigate whether there was a relationship between the spatial and directional metrics in *SpaceSwipe* and the VMI MC score, separate LMMs were fitted for each offset metric per trajectory. We used age and age squared as control variables, and the specific offset metric as a predictor to investigate the linear relationship to the VMI MC. Marginal and conditional part *R*^2^ and F-tests were used to investigate the effects of specific offset metrics.

Finally, to investigate whether there was a relationship between age and the spatial and directional metrics in *SpaceSwipe,* separate LMMs were fitted to predict the offset metrics, per trajectory, for all the completed sessions. Age and age squared were set as independent variables, but were analyzed in combination, and hereby together referred to as age. The specific offset metric, per trajectory, was set as the dependent variable. The participants were included as random effects for intercepts. Marginal and conditional *R*^2^ and F-tests were estimated to investigate the effects of age on specific offset metrics.

## Results

A total of 40 *SpaceSwipe* sessions were performed across the 12 individuals. The number of completed motor coordination tests is shown in Table 2. The total time of *SpaceSwipe*, including the preview and administration of the first horizontal line, was on average 4 min, and all except one session were below 5 min. The total time varied if the child did multiple attempts or paused for a different duration. After each level, an animation was played where the spaceship collected the stars.

**Table 2:** Completed *SpaceSwipe* and the Beery VMI MC per participant (*n* = 12)

Participant number	Completed tests		
	*SpaceSwipe*	VMI MC	Both
1	4	4	4
2	4	4	4
3	4	4	4
4	4	4	4
5	4	4	4
6	4	4	4
7	4	4	4
8	3	3	3
9	3	3	3
10	3	2	2
11	2	0	0
12	1	1	1
Total	40	37	37

A total of 347 trajectories were performed, including all sessions and levels. One participant did not perform *zigzagC* once and another one did not perform *spiralC* once. Another participant did not perform *zigzagC* once and another did not perform *spiralC* once. One participant was excluded from the VMI comparison, due to not being able to perform the VMI MC, but was able to perform *SpaceSwipe*. The final sample for the comparison, therefore, consisted of 11 participants, 5 girls, and 6 boys, (median age 10 years [range 5–14 years]) in a total of 37 sessions.

The percentiles for motor skills were estimated from the scores of the VMI MC, which indicated that 8/11 participants scored below the 50th percentile; 7/11 had below the 25th percentile, a suggested cut-off point for below-average performance (Beery 2010; Lahav et al. 2013; Valverde et al. 2020). Thus, the majority of children tested in our sample showed difficulties with motor coordination (median = 20th percentile, range: 0–60th percentile). Scores were, at initial testing, in a median of 20 points and ranged from 7 to 26 points.

### Stability and reproducibility of SpaceSwipe metrics

#### Initial testing

**Fig. 4:**
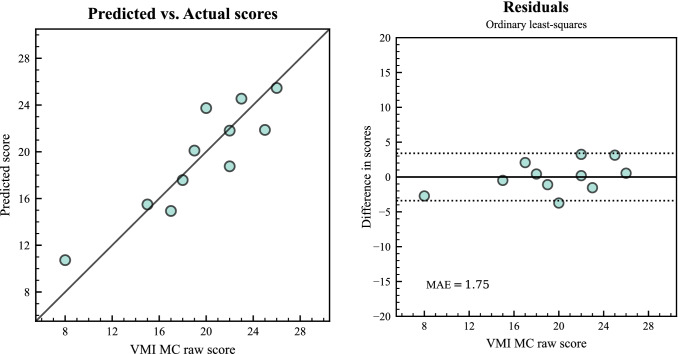
Left Panel: The predicted and the actual VMI MC score for initial testing. The solid line represents optimal alignment. Right panel: Residuals from predicting the VMI MC score (VMI score—predicted score), dotted lines represent 1.96 * SD, for the differences in scores. The distance from the solid line at 0 is how close the prediction was for that score. Mean absolute error (MAE) in the lower-left corner. *n* = 11

Results from the bivariate linear regression indicated that there was a collective significant effect between the directional offsets and the VMI MC score, *F*(2, 8) = 16.55, *p* < 0.001, *R*^2^ = 0.81, *d* = 4.01, 95% CI [2.84, 8.02]. The individual predictors were examined further and indicated that the directional offset for *spiralA* was a significant predictor for the VMI MC score, *F*(1,8) = 17.76, *p* = 0.003, *d* = 2.58, 95% CI [1.12, 4.10]. The directional offset for *zigzagA*, showed a trend but was not significant, *F*(1,8) = 4.03, *p* = 0.08, *d* = 1.10, 95% CI [0.00, 2.40]. The mean absolute error (MAE) in predicting the VMI MC score was 1.75 points (range: 0.19–3.74, see Fig. 4 for the predictions vs. actual scores and a residual plot, that displays the difference between the two scores).

When controlling for age, the two *SpaceSwipe* predictors together accounted for 53% of the variance of the VMI MC score, (95% CI [40, 66]). To account for the number of predictors, an adjusted correlation coefficient was calculated as described by Howell (2010). Based on *r* = 0.90, 95% CI [0.82, 0.97], the resulting adjusted correlation coefficient (*r*_adj_) of 0.87 suggested a strong positive correlation, and large effect size (*d* = 3.90), between our predictions and the VMI MC score.

Taken together, these findings answered the first part of our first research question RQ1 and confirmed that the offset metrics to the moving target in *SpaceSwipe* could predict the VMI MC score for the initial testing sessions.

#### Repeated testing

**Fig. 5:**
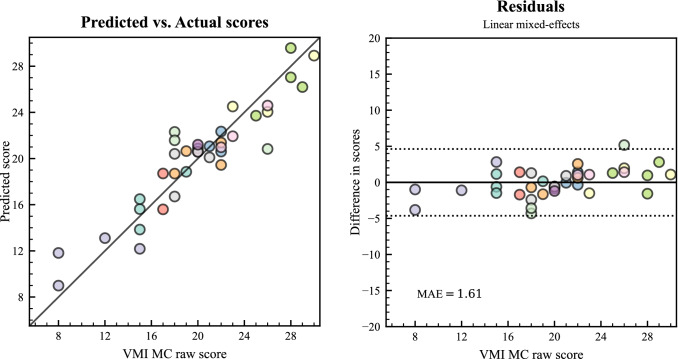
Left Panel: The predicted and the actual VMI MC scores for repeated testing. The solid line represents optimal alignment. Right panel: Residuals from predicting the VMI MC score (VMI score—predicted score), dotted lines represent 1.96 * SD, for the differences in scores. Each color represents one participant, *n* = 11. The full sample included 37 sessions. SD and MAE were estimated with equal weight per participant

All 37 mutual tests with *SpaceSwipe* and VMI MC were included from those 11 participants who performed both tests. Using the same two predictors in an LMM, F-tests revealed a significant main effect of the directional offsets for *spiralA*, *F*(1,33.01) = 20.02, *p* < 0.001, *d* = 1.48, 95% CI [0.86, 2.14], and for the directional offset from *zigzagA*, *F*(1,34.71) = 5.95, *p* = 0.02, *d* = 0.74, 95% CI [0.22, 1.36]. The MAE, weighted per participant, for predicting the VMI MC score was 1.61 points (range: 0.58–3.82), see Fig. 5 for predicted vs. actual scores and residual plot.

In order to control for the effect of age in predicting the VMI MC score, marginal and conditional part *R*^2^ (Nakagawa and Schielzeth 2013; Stoffel et al. 2021) were estimated for a model that also included age. The analyses of part *R*^2^ revealed that the directional offsets from *spiralA* and *zigzagA* accounted for more variance than age, in marginal part *R*^2^ (0.28, 95% CI [0.15, 0.44] vs. 0.16, 95% CI [0.02, 0.32]) and conditional part *R*^2^ (0.39, 95% CI [0.06, 0.59] vs. 0.27, 95% CI [0.00, 0.50]).

Finally, using only the two directional metrics from *SpaceSwipe*, the marginal *R*^*2*^ indicated that there was a strong positive association between our predicted VMI MC scores and the actual scores, marginal *R*^*2*^ = 0.68, *d* = 2.90 (95% CI [2.35, 4.96]), corresponding to a correlation of *r* = 0.82 (95% CI [0.74, 0.93]). The adjusted correlation coefficient, *r*_adj_ = 0.79, *d* = 2.58, suggested a strong positive correlation between our predictions and the VMI MC score for multiple testing, even when adjusted for the number of predictors. The conditional *R*^*2*^ also indicated a strong explanatory effect, *R*^2^ = 0.77, *d* = 3.66, 95% CI [2.40, 5.69].

Taken together, these findings answered the second part of our first research question RQ1 and confirmed that the offset metrics to the moving target in *SpaceSwipe* could predict the VMI MC score for repeated testing, indicating that the results were stable and reproducible. In addition, we observed that age is a factor that needs to be taken into consideration.

### Relationship between the spatial and directional metrics in SpaceSwipe and the VMI MC score

**Fig. 6:**
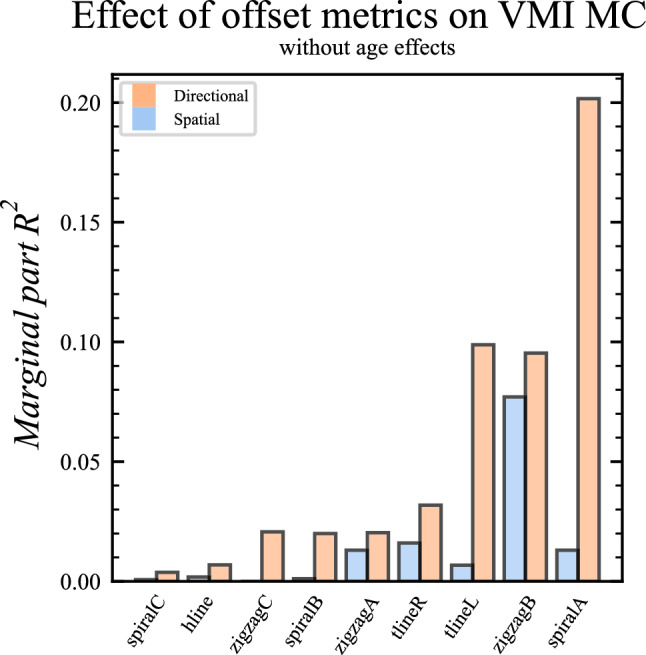
Bar plot for the difference in the marginal part *R*^2^ for the spatial and directional offset to the VMI MC score

Here, the same 37 data points from the 11 participants were used. We investigated the marginal part *R*^2^, which accounts for the effect from the specific offset, while controlling for age. The spatial offsets accounted in median for 0.2% of the variance of the VMI MC score (range: 0.0–7.7%; *d* = 0.09). The directional offsets accounted in median for 2.1% of the variance of the VMI MC score, which was numerically higher than for the spatial (range: 0.0–20.2%; *d* = 0.29). Notably, the directional offsets had numerically higher marginal part *R*^2^, for each respective trajectory, see Fig. 6.

A similar relationship was observed when investigating conditional part *R*^2^. Here, we found a median of 42% for the spatial offset (range: 39–47%; *d* = 1.70) and 40% for the directional (range: 36–44%; *d* = 1.63). The directional offsets had numerically higher conditional part *R*^2^ than the spatial in most [8 of 9] of the respective trajectories.

Furthermore, F-tests for the LMMs were highly significant for the directional offset from *spiralA* (*p* = 0.001), and borderline significant for *hlineL* and *zigzagA* (*ps* = 0.06), to the VMI MC score and insignificant for the spatial offsets, when age was included in the model.

Taken together, these findings answered our second research question RQ2 and confirmed significant relationships between directional metrics in *SpaceSwipe* and the VMI MC score, which had numerically higher marginal part *R*^2^ than the spatial metrics. This was in line with our hypothesis that the VMC MC would relate most clearly to the directional metric in *SpaceSwipe*.

### Relationship between age and the spatial and directional metrics in SpaceSwipe

**Fig. 7:**
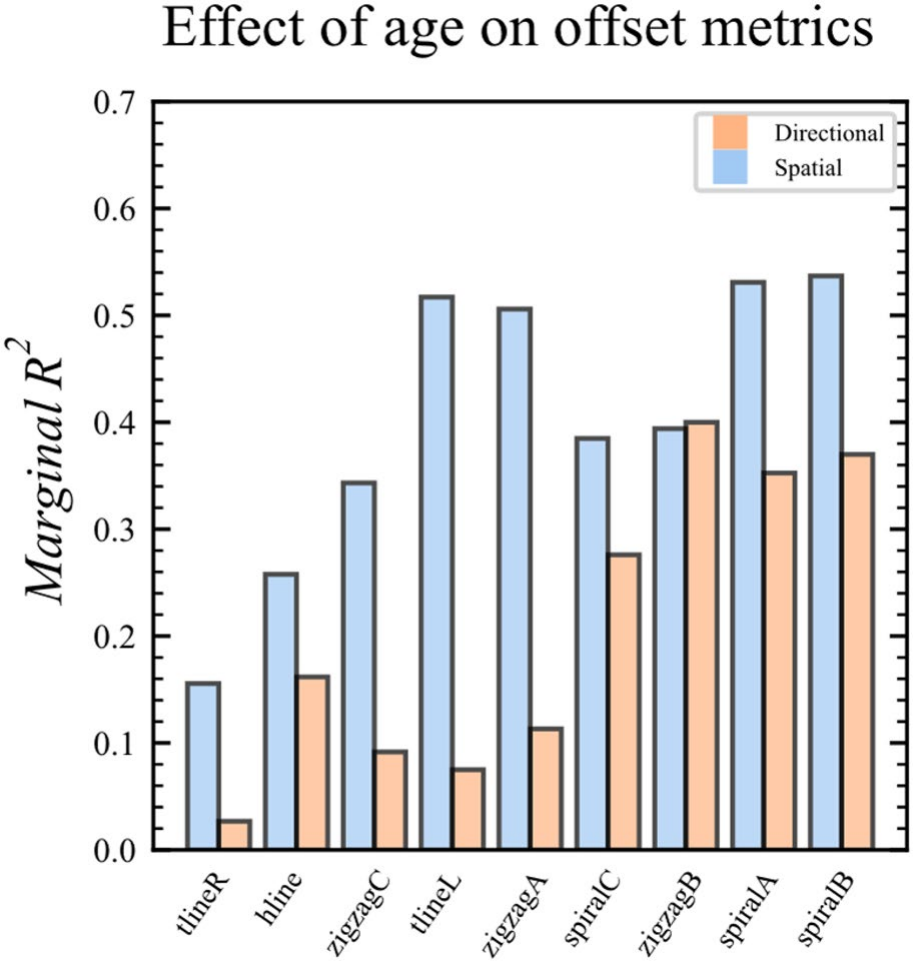
Bar plot for the difference in the marginal *R*^2^ for age to the spatial and directional offsets

Henceforth, all 40 completed *SpaceSwipe* sessions by the 12 participants were included*.* We investigated the marginal *R*^2^, to determine accounted variance for age to particular offset metrics. Age accounted in median for 39% of the variance of the spatial offset metrics (range: 16–54%; *d* = 1.60), and only 16% of the variance of the directional offsets (range: 3–40%; *d* = 0.87). The spatial offsets had numerically higher marginal *R*^2^ than the directional for all respective trajectories except for *zigzagB*, see Fig. 7.

When investigating conditional *R*^2^, which included the random effects, we found a median of 45% for the spatial offset (range: 15–72%; *d* = 1.81) and 43% for the directional offset (range: 16–65%; *d* = 1.74). The spatial offsets had numerically higher conditional *R*^2^ than directional for most of the trajectories [6 of 9].

Furthermore, separate F-tests revealed significant linear relationships between age and each metric, which were all highly significant to all the spatial offsets scores (*p*s < 0.001, for both age and age squared), and were mostly significant to the directional offsets (*p*s ≤ 0.01, except for age squared from the horizontal line [*p* = 0.11]). As mentioned, the results are reported based on all 40 *SpaceSwipe* sessions but remained consistent when only including the data when both VMI MC and *SpaceSwipe* were performed (*n* = 37).

Taken together, these findings answered our third research question RQ3 and confirmed significant relationships between age and the spatial metrics from *SpaceSwipe,* which in general had numerically higher marginal *R*^2^ than age to the directional metrics.

## Discussion

In this study, we tested motor coordination in a population of children with a specific neuropsychiatric syndrome, PANS, using both *SpaceSwipe,* a novel tablet-based motor coordination test*,* and the traditional VMI MC. We found that *SpaceSwipe* could predict VMI MC scores with high accuracy and provide temporal metrics related to motor development. Our results demonstrate that the *SpaceSwipe* motor coordination test, by using directional metrics from two trajectories, was able to accurately predict motor coordination scores obtained by VMI (*r* = 0.90, 95% CI [0.81, 0.96], *r*_adj_ = 0.87). Of note, several of our obtained correlations with VMI MC are of similar magnitude as the reported test–retest correlation for the VMI MC [*r* = 0.85, *n* = 142, with an average of 14 days between testing (Beery 2010)] and in studies investigating concurrent validity of the VMI MC [e.g., the Comprehensive Tests of Basic Skills [*r* = 0.65, *n* = 122] (Beery 2010)].

When investigating repeated testing, a strong correlation between the two measures was also identified (*r* = 0.82, 95% CI [0.74, 0.93], *r*_adj_ = 0.79), which is in line with previous findings for concurrent validity (Beery 2010).

To obtain a more careful estimate of the correlation coefficient in our limited sample size (Fisher 1915), we included a total of 37 data points derived from the participants’ sessions, thereby including more individual variances (Schober and Vetter 2018). Moreover, we used the marginal *R*^2^ to calculate correlations, which we adjusted for degrees of freedom (Howell 2010). Additionally, throughout the analysis, we controlled for the effect of age and considered both the effects of age and age squared. Nonetheless, our reported effect sizes should be interpreted with caution, as they were based on a small cohort.

In our analysis of specific offset metrics, when removing the effects of age, we confirmed a significant linear relationship between the directional offset and the slowest spiral in *SpaceSwipe* to the VMI MC score (*p* = 0.001). This relationship was not significant for any of the other spatial offsets that also had numerically lower marginal part *R*^2^ for the VMI MC score than the directional offsets, for all trajectories. The directional offset from the slowest spiral also accounted for the most variance in the VMI MC score. One potential explanation is that since the directional offset does not require an exact spatial position, it is expected to be less temporally challenging, thus being more similar to scores obtained with the VMI MC.

The findings that the directional offsets were numerically higher in marginal part *R*^2^ to the VMI MC score than to the spatial offsets, were in line with our hypothesis, considering the similarities in maintaining and adjusting the direction in tracing a path with a pencil in the VMI compared with following the direction of the moving target in *SpaceSwipe*.

In our analysis of specific offset metrics and age, we found significant linear relationships between age and the spatial offset metrics from all the trajectories. This relationship was also significant for the directional offset metrics from most of the trajectories. Age had numerically stronger associations, according to marginal *R*^2^, to the spatial metrics for all *SpaceSwipe* trajectories.

The relationship between age and both spatial and directional offset metrics of *SpaceSwipe* is not surprising, as both relate to motor development, and corresponds to the findings in preschool children tracking a moving target with a stylus by Flatters et al. (2014) and Hill et al. (2016). This relationship could stem from the development of finger-force coordination (Shaklai et al. 2017; Shim et al. 2007). Marginal *R*^2^ was in general numerically higher for spatial offsets to age than for the directional offsets. The strong relationship between age and the spatial offset is an indication that the spatial offset is a promising metric for the continuous temporal accuracy of motor coordination.

*SpaceSwipe*’*s* ability to capture temporal features related to fine motor coordination is, therefore, superior to that of the VMI MC and the Beery VMI, which previously were shown to not be correlated, or weakly correlated, to temporal features of handwriting (Brown and Link 2015; Rosenblum et al. 2019).

### General technical aspects and limitations

Several technical aspects concerning *SpaceSwipe* are important to consider, in terms of strengths and limitations. First, unlike other assessment apps (Anzulewicz et al. 2016; Chua et al. 2022; Culmer et al. 2009; Howe et al. 2017; Matic and Gomez-Marin 2019), one of the strengths of *SpaceSwipe* is that it is performed on a Raspberry Pi-based system, which ensures that the data will not be affected by unexpected operating system updates or varying components of commercial systems such as Apple iOS (Passell et al. 2021). A practical limitation is that our custom device needs to be 3D printed and assembled manually. In addition, our device is not wireless but is powered with an AC power cord, which could make portability more difficult. However, since the *SpaceSwipe* device was designed to be fixed to the table this did not hinder the experimenter or the children.

Moreover, our tracking task differs from previous studies that have included the task of tracking a moving target [c.f., Culmer et al. (2009); Flatters et al. (2014); Hill et al. (2016); Snapp-Childs, Flatters, Fath, Mon-Williams, and Bingham (2014)], as we included the power-law relationship between speed and curvature. The use of the power-law relationship might be beneficial for identifying motor difficulties as it favors smooth movement (Gulde and Hermsdörfer 2018) and increases the dynamic complexity that may be more challenging for children with DCD than without (Bo et al. 2008).

Alterations of basal ganglia function have been linked to motor difficulties in PANS (Zheng et al. 2020). Several elements of *SpaceSwipe*, such as the continuous corrective process of tracking the spaceship, involve reciprocal patterns of movement initiation and termination, and the inhibition of extemporaneous movements, which are known to be supported by basal ganglia function (DeLong and Wichmann 2007; Kennard 1944; Kim and Hikosaka 2015). Future research would benefit by directly further exploring the neurobiological signature of motor problems in PANS.

Nevertheless, several differences between the traditional, pen-and-paper-based VMI MC task and the *SpaceSwipe* are important. *SpaceSwipe* is not a tracing task like the VMI MC, as it involves active tracking of a moving target. And importantly, no stylus or pencil was used in *SpaceSwipe*, removing the need for time and practice to obtain a proper grasp and precision with a stylus or pencil (Forssberg, Eliasson, Kinoshita, Westling and Johansson 1995; Lin et al. 2017). Gaul and Issartel (2016) suggest the use of touch-screen technology may be more beneficial and more representative of the motor skills of young children, as it removes the need for pencil proficiency (Gerth et al. 2016). For these reasons, to make it more applicable across ages and varying levels of development, we decided to not use a stylus for *SpaceSwipe*.

Further, *SpaceSwipe* is faster to complete, with the full task being slightly shorter than the VMI MC subtest (< 4 min compared to 5 min, plus manual scoring, and demonstration), and with only 33 s of active movement data being sufficient for the prediction. In the future, the full *SpaceSwipe* task could be further abbreviated by excluding the fastest trajectories at 350 pix/s (0.57 cm/s), since these trajectories were too fast for some participants. Additionally, no manual scoring or extensive education was required to evaluate motor coordination with *SpaceSwipe*, which further reduces total test duration and improves ease of administration. *SpaceSwipe* has a higher spatial resolution than the VMI MC, based on comparing the pixel size of 0.2 mm to the minimum outline path of 4.5 mm in the VMI MC. This suggests that *SpaceSwipe* can assess spatial aspects of motor control with greater precision and that, together with its temporal dimension measured at 60 Hz, thoroughly captures the spatiotemporal aspects of motor coordination with greater sensitivity.

Nonetheless, the major limitation of the present study is that our results are based on findings obtained in a small, yet heterogenous, group of 12 children with PANS, of whom 11 were compared to VMI MC scores. Thus, this study demonstrates proof-of-concept of this new methodology, with pilot results indicating its excellent practical potential. Further work is now required to assess larger groups consisting of children with PANS, children with other neurodevelopmental conditions, as well as children from the general population, in order to obtain normative data for these novel metrics, and to compare them against validated instruments such as the Beery VMI and MABC-2 (Beery 2010; Henderson et al. 1992) and in relation to specific neuropsychiatric symptoms. Importantly, our group of children with PANS represents a relatively rare population of individuals with a specific neuropsychiatric disorder that affects, among other functions, motor coordination. We aimed to examine how our approach could help better characterize and follow-up their unique motor difficulties. Another limitation is that previous exposure to tablets and pencil writing, which could be potential confounding factors, were not collected, and we suggest that they should be considered in future research.

Finally, our data were based on a group of children with PANS, which means that the results may or may not be generalizable to other pediatric or adult populations. The participants had several possible causes of their motor difficulties. Nearly half of them had ADHD, almost all had motor hyperactivity, and over half had described dysgraphia. Nevertheless, it is notable that significant linear relationships between the offset metrics to the VMI MC scores were identified in the most conservative analysis using repeated testing, when controlling for age, in our relatively small sample including effects of age and overall functioning level, giving some considerable promise of the capability of *SpaceSwipe* as a practicable and sensitive motor measure for children.

## Conclusions

*SpaceSwipe* provided an objective reference of the children’s visuomotor performance, giving a direct computational metric of motor control. *SpaceSwipe* results predicted Beery VMI scores in a shorter time than the Beery itself, and with high accuracy. Directional offsets accounted for the large variance of the VMI MC score, and in contrast, spatial offsets appeared related to age. In sum, *SpaceSwipe* offers novel directional and spatial offset metrics to a target movement and provides a continuous assessment of movement quality. The findings from this proof-of-concept study demonstrate that *SpaceSwipe* is a promising tool for motor testing, giving additional temporal measures of children’s motor control within complex symptomatic conditions that are accompanied by clinical difficulties in coordinating and controlling movement.

## Supplementary Information

Below is the link to the electronic supplementary material.

Supplementary Methods (PDF 554 KB)

Supplementary Tables (PDF 188 KB)

## Data Availability

None of the studies reported in this article were preregistered. The data have not been made available on a permanent third-party archive because participants were not asked to consent for their data to be made publicly available, even anonymized. Data are available upon request from those who wish to collaborate with us, via a Visitor Agreement with the University of Gothenburg, if appropriate, under existing ethical approval. Detailed steps of the procedures for generating stimulus, including code, are available in the Supplemental Material associated with this article.
