# Supplementary material for: A novel tablet-based motor coordination test performs on par with the Beery VMI subtest and offers superior temporal metrics: findings from children with pediatric acute-onset neuropsychiatric syndrome
Source: Exp Brain Res. 2023 Apr 13;241(5):1421–36. doi: 10.1007/s00221-023-06612-x (PMC10130113; doi:10.1007/s00221-023-06612-x)
Supplement: Supplementary file 1 — Supplementary Methods (PDF 554 KB) [file 221_2023_6612_MOESM1_ESM.pdf]

Supplementary Methods

Mathematical operations and Python implementations

M. Thorsson et al.

This supplementary text serves as an extended method to the paper, *A novel tablet-based motor coordination test performs on par with the Beery VMI subtest and offers superior temporal metrics: findings from children with Pediatric Acute-onset Neuropsychiatric Syndrome* (PANS). Mathematical operations and Python 3.8.5 (Van Rossum and Drake Jr, 1995) implementations, used in the present study, for path and motion profile generation, and estimation of offset metrics for the *SpaceSwipe* motor coordination test, are outlined here.

1 Path generation

1.1 Mathematical operations

We describe the mathematical operations used to create the paths, in four parts (1, 2a, 2b, and 3). In *Part 1*, a scaling transformation along the path is optionally added. *Part 2a* is chosen to create a path with a tangential angle that has a constant direction (either clockwise or counterclockwise), as in a spiral. *Part 2b* is chosen to create a path with a tangential angle that has an alternating direction, as in a zigzag (that alternates between clockwise and counterclockwise direction). In *Part 3*, the coordinates of the paths are obtained.

Part 1

To obtain a scaling along the path, as in making the radius of a spiral smaller and smaller, an array with equally spaced numbers, from 0 to 1 (x) is transformed by a normalized tunable sigmoid (previously presented by Dini (2010)).

Let k_d be the degree of curvature along the path, ranging from -1 to 1.

Then the scaling (x_1) can be obtained by the equation

$$x_1 = \frac{x - k_d x}{k_d - 2k_d|x| + 1}.$$

Let f be the shift parameter, ranging from -0.5 to 0.5.

Let c be the number of turns.

Then, by using the modulo operator (%), we can create an array (x_2) suitable for the repetition of a normalized tunable sigmoid.

$$x_2 = (x_1 2c + 2f) \,\%\, 2 \; - 1.$$

Part 2a

This part is for generating paths with constant direction, as in a spiral.

Let x_2 be the transformed x obtained from *Part 1*.

Let k be the degree of curvature of the turns, ranging from -1 to 1.

x_2 is then transformed using the normalized tunable sigmoid function, so that

$$s_{sig} = \frac{x_2 - kx_2}{k - 2k|x_2| + 1}.$$

The step-wise ceiling, x_{ceil}, makes it possible to adjust s_{sig}, to a smoothly increasing sequence and is obtained by the equation

$$x_{ceil} = 2\lceil \frac{x_1 c + f}{2} \rceil.$$

The sum of s_{sig} and x_{ceil} is then multiplied by twice the amount of rotation (d), to obtain the tangential angle (φ).

$$\varphi = \frac{2d}{c}(s_{sig} + s_{ceil}).$$

Part 2b

For a path that changes the direction of rotation, as in a zigzag, *Part 1* is first performed.

Let x_2 be the transformed x obtained from *Part 1.*

Let k be the degree of curvature of the turns, ranging from -1 to 1.

x_2 is then transformed using the normalized tunable sigmoid function, so that

$$s_{sig} = \frac{x_2 - kx_2}{k - 2k|x_2| + 1}.$$

An array that can flip every second normalized tunable sigmoid, x_{flip}, is obtained by the equation

$$x_{flip} = 2\left\lfloor \frac{(x_1 c + f)\,\%\,2}{2} \right\rceil - 0.5.$$

Finally, to obtain the tangential angle (φ), x_{flip} is multiplied by s_{sig} and twice the amount of rotation (d) divided by the number of turns (c):

$$\varphi = x_{flip} s_{sig} \frac{2d}{c}.$$

Part 3

The x- and y-coordinates are obtained from the cumulative sum of the cosine respective sine of φ (from either *Part 2a* or *Part 2b*). This way, each step along

the path represents a unit vector in the direction of the tangential angle.

$$xpos\,[i] = \sum_{n=1}^{i} cos(\varphi\,[n]),\ \text{for } i = 1, 2, \ldots, N.$$

$$ypos\,[i] = \sum_{n=1}^{i} sin(\varphi\,[n]),\ \text{for } i = 1, 2, \ldots, N.$$

Examples for respective, arrays and resulting paths with constant direction are displayed in Fig. 1 and for alternating direction in Fig. 2.

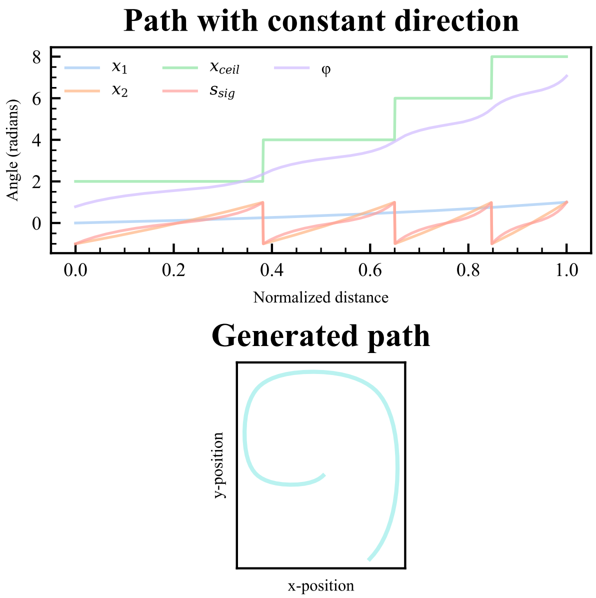

Fig. 1: Example generation of a path with constant direction. See the upper figure for the transformed arrays (upper) and for the generated paths (lower) using the parameters $c = 4, d = 4\pi, f = 0.5, k = 0.4$, and $k_d = 0.3$. The terminology corresponds to the previously introduced.

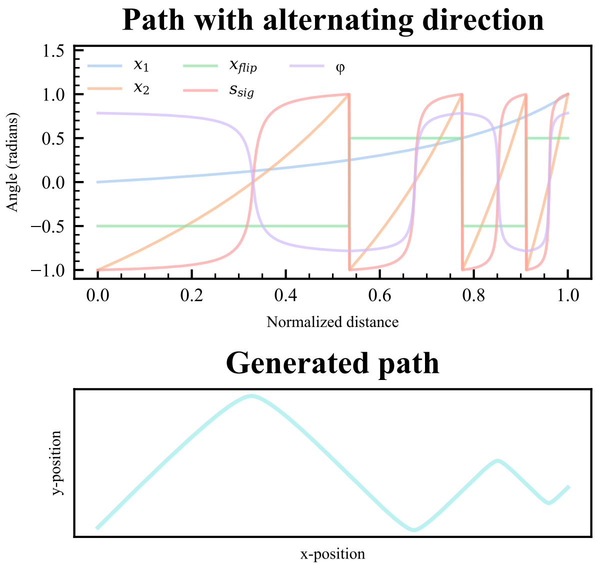

Fig. 2: Example generation of path with alternating direction. See the upper figure for the transformed arrays and lower for the generated paths using the parameters $c = 7, d = 4\pi, f = 0, k = -0.4$, and $k_d = 0.3$. The terminology corresponds to the previously introduced.

1.2 Python implementation

We here present our implementation of the path generation in Python 3.8.5 examples. Several of the functions have a dependency on the NumPy library (Harris et al., 2020), version 1.20.1. First, we define the functions needed for the path generation.

Normalized tunable sigmoid

The normalized tunable sigmoid can be expressed in a Python function as follows:

```
import numpy as np#import NumPy library
nf=np.finfo(float).eps#a very small number, to prevent division by zero
def normalized_sig(x,k):
   return (x-x*k)/((k-abs(x)*2*k+1)+nf)
```

Tangential angle

To generate the tangential angle for the paths, we define three Python functions. First, we create a function for paths with a constant direction:

```
def spiral_func(x1,c,f,k,d):
    x2=(x1*2*c+2*f)%2-1#array for repetition of normalized tunable sigmoid
    xc=np.ceil(x1*c+f+nf)*2#stepwise ceiling
    ssig=(normalized_sig(x2,k))#repeated normalized tunable sigmoids
    return (ssig+xc)/2*d/c#make constant stepwise and scale to rotation
```

Second, we define a function for paths with alternating direction:

```
def zigzag_func(x1,c,f,k,d):
    x2=(x1*2*c+2*f)%2-1#array for repetition of normalized tunable sigmoid
    xf=np.round(((x1*(c)+f)%2)/2)-0.5#flipping array
    ssig=(normalized_sig(x2,k))#repeated normalized tunable sigmoids
    return ssig*xf*d/c#flip normalized sigmoids and scale to rotation
```

Finally, a function to transform the tangential angle to 2D coordinates, and that scales to screen size are defined:

```
w,h=800,480#screen size
def get_xy(t_g):#obtain coordinates, scale and centering
    xpyp=np.array([np.cumsum(np.cos(t_g)),np.cumsum(np.sin(t_g))])#
     cumulative sum of cos and sin
    s=np.max(xpyp,1)-np.min(xpyp,1)#path dimensions
    s2=([w,h][s.argmax()])/max(s)
    xpyp*=((0.8*[w,h][s.argmax()])/max(s))
    s=np.max(xpyp,1)-np.min(xpyp,1)
    if min(s)>[w,h][s.argmin()]:
        xpyp*=((0.8*[w,h][s.argmin()])/min(s))
    xpyp-=np.mean(xpyp,1).reshape(-1,1)#center to figure center
    return xpyp+np.array([[w/2,h/2]]).T#centering
```

Example path generation

Here we show one example of how to create a path with constant direction (spiral) and one for alternating direction (zigzag). Both types of paths require

an array (x) of discretely spaced numbers ranging from 0 to 1, which can be obtained using the NumPy library (Harris et al., 2020) as follows:

```
x=np.linspace(0,1,5000)
```

The following parameters and functions can be used to create a spiral path.

```
#spiral generation
c=11#number of turns
f=0#shift parameter
k=0.4#degree of curvature
d=6*np.pi#amount of rotation
kd=0.3#degree of curvature along the path
x1=normalized_sig(x,kd)#scaling along the path
t_ang=sprial_func(x1,c,f,k,d)#get tangential angle
x,y=get_xy(t_ang)#get spiral path
```

The same works for a zigzag path, but preferably with a negative k and a lower amount of rotation:

```
#zigzag generation
k=-0.9#degree of curvature
d=5*np.pi#less amount of rotation
t_ang=zigzag_func(x1,c,f,k,d)#get tangential angle
x,y=get_xy(t_ang)#get zigzag path
```

In Fig. 3 the generated paths are displayed.

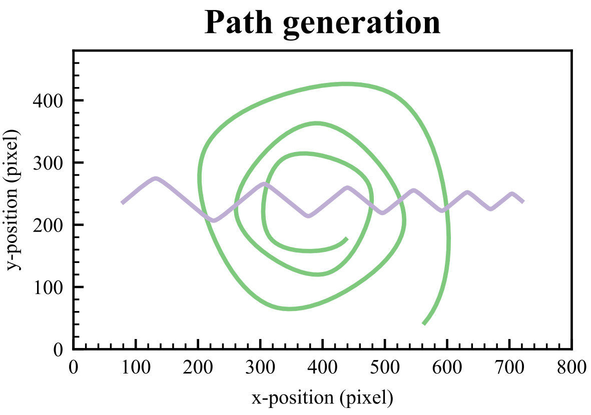

Fig. 3: Generated spiral (green) and zigzag (purple) paths.

2 Motion profile generation

The motion profile for the horizontal line was based on the minimum-jerk trajectory. The minimum-jerk trajectory is a smooth trajectory obtained by minimizing the jerk (change of acceleration) between two positions, which has been proposed to be crucial for human upper limb movement (Flash and Hogan, 1985).

The tilted lines were at a constant speed. The other motion profiles of the gestures are based on the principle of a power-law relationship (Lacquaniti, Terzuolo, and Viviani, 1983) between curvature and speed. The tuning parameter β, was set based on the findings by Huh and Sejnowski (2015).

2.1 The minimum-jerk trajectory

2.1.1 Mathematical operations

Here, the mathematical operation to obtain the position of the minimum-jerk trajectory is presented.

Let d be the travel time and t the time step, then the position at the minimum-jerk trajectory is obtained by the equation

$$x(t) = x_i + (x_f - x_i)\left(10\left(\frac{t}{d}\right)^3 - 15\left(\frac{t}{d}\right)^4 + 6\left(\frac{t}{d}\right)^5\right),$$

where x_f is the final position and x_i is the current position.

2.1.2 Python implementation

Here we present the steps in Python, to create a minimum-jerk trajectory for a horizontal line.

The position in a minimum-jerk trajectory, for a sequence of time steps with a constant frame rate, can be implemented in the Python function:

```
def mjt(steps):#number of time steps
```

```
    s1=steps+1
    x=np.arange(1,s1)
    return 10*(x/s1)**3-15*(x/s1)**4+6.0*(x/s1)**5
```

The number of time steps can be estimated based on the target average speed, total distance, and frame rate. Then, the minimum-jerk trajectory can be obtained, as follows:

```
distance=600#total distance of the trajectory
tv=140#the target average speed (pixel/s)
hz=60#frame rate
steps=int(np.around((distance/tv)*hz))#converted to total datapoints
mjt_pos=np.array(mjt(steps))*distance#scaled by distance
mjt_pos=mjt_pos+(w-distance)*0.5#center to screen
```

The generated displacement function is displayed in Fig. 4.

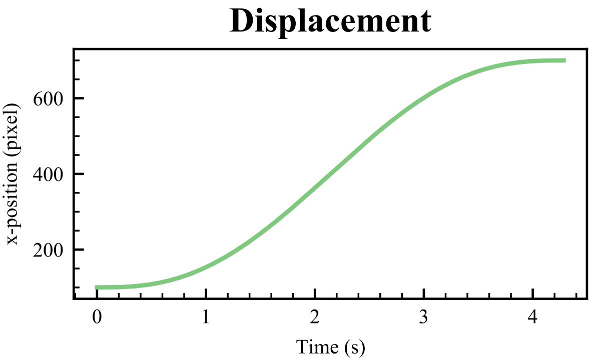

Fig. 4: The displacement profile of the generated minimum-jerk trajectory.

The generated speed function is displayed in Fig. 5, which also shows the resulting average speed.

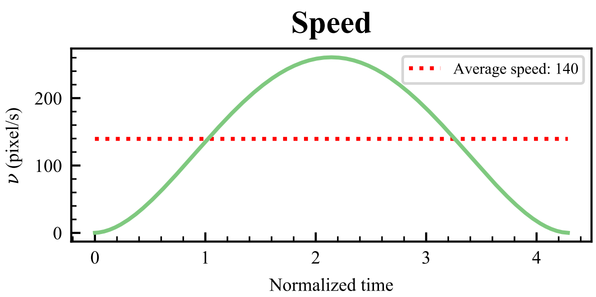

Fig. 5: The speed profile of the generated minimum-jerk trajectory.

The normalized speed, from the minimum-jerk trajectory, is visualized along the path, with increasing color intensity and width, by speed, in Fig. 6.

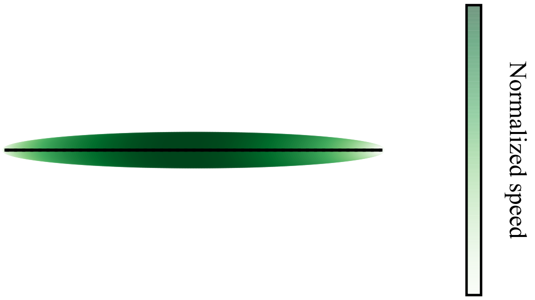

Fig. 6: The speed, obtained from the minimum-jerk trajectory, visualized along the path.

2.2 Power-law relationship

2.2.1 Mathematical operations

The motion profiles, for the spiral and zigzag paths, were based on a power-law relationship between curvature and speed (Lacquaniti et al., 1983), following the principles presented by Huh and Sejnowski (2015).

Let κ denote the curvature and ν, the speed.

Let β be the exponent, which tunes the relationship.

The power-law relationship, for obtaining the speed profile is expressed by the equation

$$\nu = \kappa^{-\beta}.$$

Tangential angle and curvature

The tangential angle is the direction of the tangential velocity along the path. Since we assume a constant frame rate, the tangential angle can be estimated using the 2-argument arctangent ($arctan2$) of the derivatives of the x- and y-position.

$$\varphi = arctan2(x', y').$$

Curvature can be interpreted as the inverse radius of the osculating circle of a curve at a given point. Here, the signed curvature (κ), is a function of time (at constant frame rate) and can be estimated using the first and second order derivatives (velocity and acceleration) of the x- and y-position.

$$\kappa = \frac{x'y'' - y'x''}{(x'^2 + y'^2)^{\frac{3}{2}}}.$$

2.2.2 Python implementation

We first create a Python function used to estimate the tangential angle:

```
def get_tan(pts):#estimate tangential angle
    dx_dt,dy_dt=np.gradient(pts[0]),np.gradient(pts[1])
    return np.arctan2(dx_dt,dy_dt)
```

Since we are using a constant frame rate, the time delta can be excluded. For convenience, we use accurate central differences as implemented in NumPy (Harris et al., 2020).

The same approach can also be used for estimating curvature:

```
def get_curvature(pts):#estimate curvature
    dx_dt,dy_dt=np.gradient(pts[0]),np.gradient(pts[1])
    d2x_dt2,d2y_dt2=np.gradient(dx_dt),np.gradient(dy_dt)
    return (d2x_dt2*dy_dt-dx_dt*d2y_dt2)/(dx_dt*dx_dt+dy_dt*dy_dt)**(3/2)
```

As well as for estimating speed, which we here define in a Python function:

```
def get_speed(pts):#estimate speed
    dx_dt,dy_dt=np.gradient(pts[0]),np.gradient(pts[1])
    return np.sqrt(dx_dt*dx_dt+dy_dt*dy_dt)
```

We then use the points from the, previously created, zigzag and estimate the curvature. The tangential angle (see Fig. 7) and the curvature (see Fig. 8) can then be estimated as follows, using the coordinates from the previously generated zigzag path:

```
pts=np.array([x,y])#array of x- and y-coordinates
tan=get_tan(pts)#estimate tangential angle
k=get_curvature(pts)#estimate curvature
```

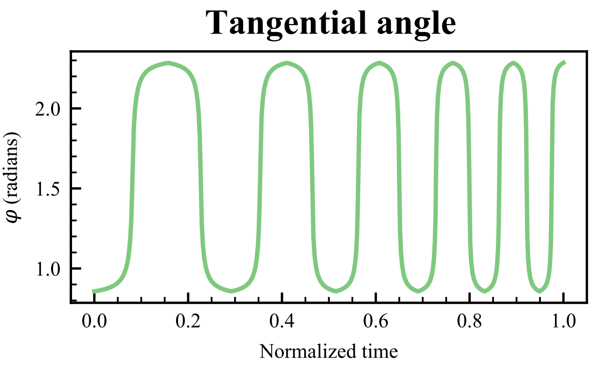

Fig. 7: Estimated tangential angle of the generated path.

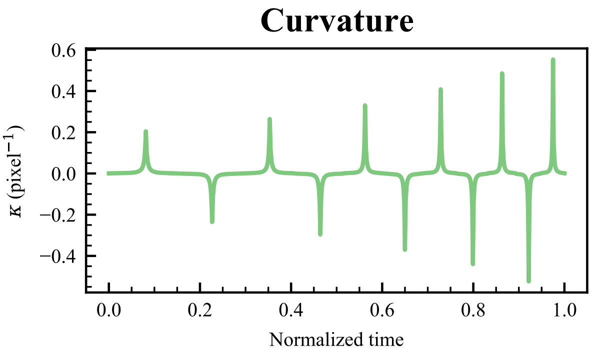

Fig. 8: Estimated curvature of the generated path.

Obtaining the displacement profile

A linear interpolator can be used to create a function, for example, $f(x) = y$, when x and y are known. Then, new values of the function can be estimated based on linear interpolation. We use linear interpolation as implemented in the SciPy library (Virtanen et al., 2020), version 1.8.1. Two linear interpolators are created. First, an interpolator, that estimates the inverse cumulative sum of the calculated speed based on a constant frame rate is created. This generates our displacement profile, based on our desired number of data points. Second, an interpolator that provides the coordinates of the path based on the displacement is made.

We start by defining a Python function, to estimate the distance along the path:

```
def get_d(pts):#function to estimate displacement
    distance=np.cumsum(np.sqrt(np.sum(np.diff(pts.T,axis=0)**2,axis=1)))
    return np.insert(distance,0,0)
```

Followed by creating the Python function to obtain the coordinates based on the speed profile:

```
from scipy.interpolate import interp1d
def get_equi(pts,vi,tp):#get coordinates based on speed profile
    distance=get_d(pts)
    distance=distance/max(distance)#scale to 0-1
    alpha1=np.linspace(0,1,len(vi))
    alpha2=np.linspace(0,1,tp)
    interpolator2=interp1d(vi,alpha1,fill_value='extrapolate')#for displacement profile
    interpolator1=interp1d(distance,pts.T,axis=0,fill_value='extrapolate')#for coordinates based on displacement profile
    return interpolator1(interpolator2(alpha2))
```

Finally, the trajectory can be obtained by passing the chosen number of equally spaced points through the first interpolator, and that result is then passed through the second interpolator. Altogether, this can be implemented as follows:

```
b=0.3#beta
v=abs(k)**-b#speed accordint to power-law
d=get_d(pts)#distance along path
vi=np.cumsum((v+nf)**-1)#the cumulative sum of the inverse speed
vi=vi/max(vi)#normalization
tv=100#the target average speed (pixel/s)
ptd=max(d)/tv#points in total distance
tp=int(hz*ptd)#number of points
xy=get_equi(pts,vi,tp)#get coordinates
v2=get_speed(xy.T)*hz#estimate speed
```

In Fig. 9 the generated speed profile is displayed, which slows down with increasing curvature. The normalized speed is visualized along the path, with increasing color intensity and size with speed, in Fig. 10.

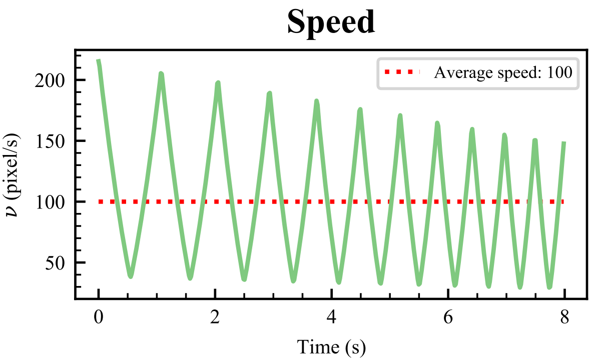

Fig. 9: The speed profile of the generated trajectory based on the power-law relationship between curvature and speed.

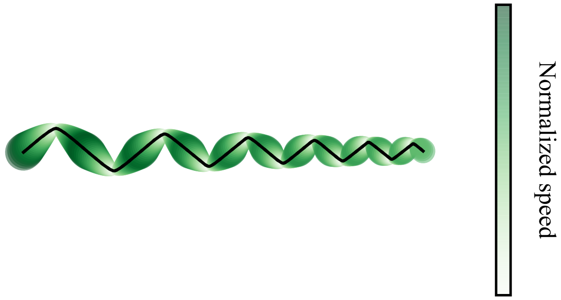

Fig. 10: The normalized speed, obtained from the power-law relationship between curvature and speed, visualized along the path.

Confirmation of power-law relationship

To confirm the power-law relationship, we perform a robust linear regression, with a Huber M-estimator using the Statsmodels library (Seabold and Perktold, 2010), version 0.13.2. Both the speed and curvature are log-transformed.

```
v2=get_speed(xy.T)*hz#estimate speed
k2=abs(get_curvature(xy.T))#estimate curvature
#Huber regression
import statsmodels.api as sm
rlm_model=sm.RLM(np.log(v2+nf), sm.add_constant(np.log(k2+nf)), M=sm.robust
    .norms.HuberT())
rlm_results=rlm_model.fit()
print(rlm_results.summary().tables[1].as_latex_tabular())
```

This provides us with the following Latex table output:

Table 1: Statsmodels regression output.

	**coef**	**std err**	**z**	$p>\|z\|$	**[0.025**	**0.975]**
**const**	-1.1318	0.001	-980.703	0.000	-1.134	-1.129
**x1**	-0.2981	0.000	-1416.092	0.000	-0.299	-0.298

As displayed in Table 1, the relationship between curvature, and speed from our generated trajectory, could through regression confirm that our approach provided a corresponding β. Below in Fig. 11, the regression is displayed. The outliers are caused by the discreteness of the data but were not noticeable in the tracking of the trajectory.

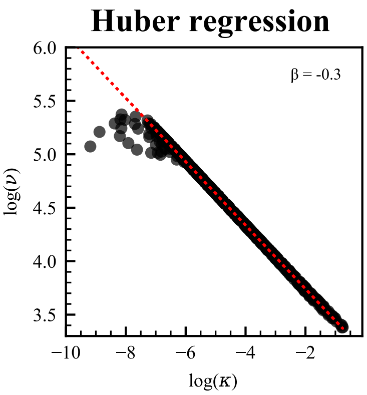

Fig. 11: Result from the Huber regression.

3 Offset metrics

Motor performance was in *SpaceSwipe* measured by offset metrics to the target movement's position and direction. We expressed the directional offset as the delta between the tangential angle of the moving spaceship and the participant's movement. Hereby, we describe how it was estimated. Let x_1, y_1 be the position that occurs at the time t_1 followed by the position x_2, y_2 at the time t_2 (the time delta is not required, in our case, but is added for clarity). Then, the tangential velocity for the x- and y-coordinates (v_x, v_y) are estimated:

$$v_x = \frac{x_1 - x_2}{t_1 - t_2}, \; and \quad v_y = \frac{y_1 - y_2}{t_1 - t_2}.$$

The tangential angle can then be estimated using the 2-argument arctangent of the tangential velocity:

$$\varphi = arctan2\left(v_x,\ v_y\right).$$

The tangential angles are estimated for the spaceship's and the participant's movements. The directional offset (φ_{offset}) is then obtained from the absolute delta, so that

$$\varphi_{offset} = |\varphi_{participant} - \varphi_{spaceship}|.$$

The directional offset, which is in radians, is then normalized to the range 0 – π, and converted to degrees before statistical analysis and visualization. We expressed the spatial offset as the distance that the participant had to the target position, the Euclidean distance between the touch position and the spaceship center, which was estimated as follows: Let x_1, y_1 be the position of the participant's finger and x_2, y_2 be the target position (the center of the

spaceship). The spatial offset (s_{offset}) is obtained by the equation:

$$s_{offset} = \sqrt{(x_1 - x_2)^2 + (y_1 - y_2)^2} .$$

References

Dini, D. (2010). Normalized tunable sigmoid functions. https://dinodini.wordpress.com/2010/04/05/normalized-tunable-sigmoid-functions/

Flash, T., and Hogan, N. (1985). The coordination of arm movements: an experimentally confirmed mathematical model. *J. Neurosci.*, *5*(7), 1688. doi: 10.1523/JNEUROSCI.05-07-01688.1985

Harris, C.R., Millman, K.J., van der Walt, S.J., Gommers, R., Virtanen, P., Cournapeau, D., ... Oliphant, T.E. (2020, September). Array programming with NumPy. *Nature*, *585*(7825), 357–362. doi: 10.1038/s41586-020-2649-2

Huh, D., and Sejnowski, T.J. (2015). Spectrum of power laws for curved hand movements. *Proc. Natl. Acad. Sci. U.S.A.*, *112*(29), E3950-E3958. doi: 10.1073/pnas.1510208112

Lacquaniti, F., Terzuolo, C., Viviani, P. (1983). The law relating the kinematic and figural aspects of drawing movements. *Acta Psychol (Amst)*, *54*(1-3), 115–130. doi: 10.1016/0001-6918(83)90027-6

Seabold, S., and Perktold, J. (2010). Statsmodels: Econometric and statistical modeling with python..

Van Rossum, G., and Drake Jr, F.L. (1995). *Python tutorial.* Centrum voor Wiskunde en Informatica Amsterdam.

Virtanen, P., Gommers, R., Oliphant, T.E., Haberland, M., Reddy, T., Cournapeau, D., ... SciPy 1.0 Contributors (2020). SciPy 1.0: Fundamental Algorithms for Scientific Computing in Python. *Nat. Methods*, *17*, 261–272. doi: 10.1038/s41592-019-0686-2
