# Supplementary material for: A novel tablet-based motor coordination test performs on par with the Beery VMI subtest and offers superior temporal metrics: findings from children with pediatric acute-onset neuropsychiatric syndrome
Source: Exp Brain Res. 2023 Apr 13;241(5):1421–36. doi: 10.1007/s00221-023-06612-x (PMC10130113; doi:10.1007/s00221-023-06612-x)
Supplement: Supplementary file 2 — Supplementary Tables (PDF 188 KB) [file 221_2023_6612_MOESM2_ESM.pdf]

# Supplementary Tables

## Participant demographics

M. Thorsson et al.

**Table 1** Participant demographics (*n* = 12).

| | **Frequency** | **Percentage** |
|---|---|---|
| **Sex** | | |
| Boys | 6 | 50 |
| Girls | 6 | 50 |
| **Comorbid diagnoses** | | |
| ADHD | 4 | 33 |
| No neurodevelopmental disorder/symptoms | 4 | 33 |
| ≥ 2 comorbid neurodevelopmental disorders/symptoms | 4 | 33 |
| Autistic-like condition | 3 | 25 |
| Autism/Asperger syndrome | 1 | 8 |
| Unspecified epilepsy | 1 | 8 |
| **Streptococcal infection before PANS onset** | | |
| Suspected | 3 | 25 |
| Verified/colonized | 2 | 17 |
| **Concomitant medication** | | |
| Selective Serotonin Reuptake Inhibitor (SSRI) | 7 | 58 |
| ADHD medication | 4 | 33 |
| Sleep medication | 5 | 42 |
| Antibiotics | 5 | 42 |
| Antipsychotics | 3 | 25 |
| Non-steroidal anti-inflammatory drugs (NSAID) | 3 | 25 |
| Antihistamines | 1 | 8 |

**Table 2** Symptoms from the PANS scale at baseline (*n* = 12).

| PANS symptom | Frequency | Percentage |
|---|---|---|
| Obsessive-compulsive symptoms | 12 | 100 |
| General anxiety | 12 | 100 |
| Emotional lability, mood swings | 12 | 100 |
| Increased irritability or aggressive behavior | 12 | 100 |
| Panic episodes | 11 | 92 |
| Change in personality | 11 | 92 |
| Difficulties in attention or learning | 11 | 92 |
| Sensory symptoms | 11 | 92 |
| Motor hyperactivity | 11 | 92 |
| Irrational fears/phobias | 10 | 83 |
| Depression with/without suicidal/self-injurious thoughts | 10 | 83 |
| Loss of academic skills (math, reading, writing) | 10 | 83 |
| Extreme tiredness or fatigue | 10 | 83 |
| Eating restriction | 9 | 75 |
| Separation anxiety | 9 | 75 |
| Behavioral regression (behavior atypical for age) | 9 | 75 |
| Sleep problems | 9 | 75 |
| Dysgraphia | 8 | 67 |
| Simple motor/vocal tics | 8 | 67 |
| Dilated pupils | 8 | 67 |
| Complex motor/vocal tics | 5 | 42 |
| Urinary symptoms | 4 | 33 |
| Hallucinations | 2 | 17 |
| Piano playing movements | 2 | 17 |
| Confusion | 1 | 8 |
